# Nasal histoplasmosis without lung involvement in an immunocompromised patient

**DOI:** 10.5935/1808-8694.20120022

**Published:** 2015-11-20

**Authors:** Michelle Manzini, Michelle Lavinsky-Wolff

**Affiliations:** a Second-year Resident Physician at the ENT Service of the Porto Alegre University Hospital (Resident Physician); b PhD in Epidemiology - Federal University of Rio Grande do Sul. Hospital de Clínicas de Porto Alegre

**Keywords:** fungi, histoplasmosis, nasal obstruction

## INTRODUCTION

Histoplasmosis is a fungal infection caused by *Histoplasma capsulatum.* It is endemic in many parts of the world, namely in the American Midwest, Latin America, and Southern Africa^1^.

In 99% of the cases the infection is self-limiting or restricted to the lungs. Liver, spleen, lymph nodes, bone marrow, skin, and mucosas may be involved in the remaining 1% of the cases^1^.

Mucocutaneous histoplasmosis may affect immunosuppressed patients, but is rare in immunocompetent subjects^1^.

Isolated nose involvement is rare and may be mistaken for malignant tumor.

This paper reports a case of an immunosuppressed patient with nasal histoplasmosis without lung involvement and aims to stress the need for attention when the diagnoses of patients with infectious nasal lesions are produced.

## CASE PRESENTATION

**Figure 1:**
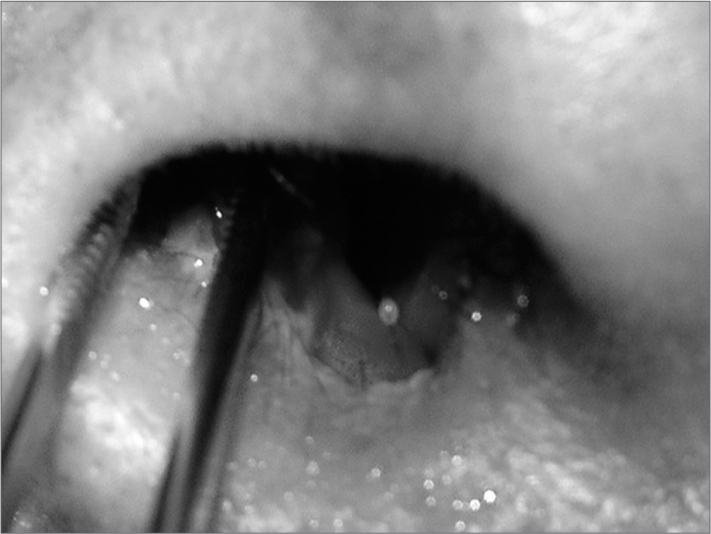
Ulcerated lesion In the patient's right nasal cavity.

JP, male, 59, retired, born and living in Porto Alegre, RS. The subject has cirrhosis due to hepatitis C and alcohol abuse, smokes, and is HIV-negative. He was hospitalized for a lower limb bypass procedure and complained of a nose wound that had been evolving for two months. The patient was examined by the ENT team and was found to have an uneven ulcerated wound covered by crust in his right nasal cavity (Figure 1). No other noteworthy findings were reported. The injury was biopsied as the the patient was initially suspected for a malignant tumor. No tests were done until the biopsy results were available.

The patient was discharged from the hospital and came back to the ENT ward with the same complaints. His physical examination showed no differences in relation to the one done prior.

The biopsy report described chronic ulcerated inflammation with granulation tissue in the respiratory mucosa. Grocott's methenamine silver (GMS) stain was positive for fungus and revealed small ovoid yeasts suggesting histoplasmosis.

The patient was further examined by the Infectology team and a chest x-ray was ordered. No signs indicative of histoplasmosis were found, and the patient was started on Itraconazole 100 mg twice a day, dosage corrected for glomerular filtration rate, for 12 months.

He was assessed one month into treatment and the nose lesions had disappeared.

## DISCUSSION

Histoplasma is ubiquitous and potentially virulent. Its spores are inhaled through the respiratory tract and, after phagocytosis and exposure to bodily temperatures, they convert into yeasts and propagate into the macrophages to reach the lymphatic system. In this stage, depending on the individual's immune status, hematogenous dissemination may occur^1^.

Infection usually starts in the lungs when the inoculum is small and the patient immunocompetent. In rare occasions the infection will spread onto other organs. When it reaches the had and neck, the most involved sites are the oral cavity, the larynx, and the pharynx. It affects only the mucosa and is frequently mistaken for malignant neoplasm^1^, granulomatous diseases, and benign nose tumors.

Due to the small size of the fungus (2-4 mm) and the similarity to other yeasts, the diagnosis of histoplasmosis is done when the fungus is isolated in a culture, in a long process that may delay diagnosis and treatment^2^.

Culture is the gold standard diagnostic test and takes from two to four weeks to provide conclusive results. However, it is not useful in severe cases when prompt intervention is required. Sabouraud agar culture is effective in 25% to 40% of the cases. In chronic aerodigestive lesions - for which the culture is not very effective - Gomori's staining elicits the infectious agent and aids in diagnosis^3^.

Systemic Itraconazole has been effective in treating patients, but it must be avoided in cases of liver disease. Amphotericin B is the drug of choice in severe or persistent infection^4^.

Surgery does not play a therapeutic role, but is useful in diagnosing patients, once histoplasmosis is not angioinvasive and extensive surgical debridement is not required^4^.

## CLOSING REMARKS

Albeit rare, histoplasmosis must be considered in the diagnosis of nose lesions, specifically in immunologically compromised patients. If culture is not done, diagnosis and treatment may be delayed to compromise the patients' prognoses.
